# Assessment of the Diagnostic Value of [^68^Ga]Ga-FAPI-04 and [^18^F]FDG PET in a PHMG-p-Induced Pulmonary Fibrosis Murine Model

**DOI:** 10.3390/diagnostics16010010

**Published:** 2025-12-19

**Authors:** So Young Kim, Jun Young Park, Ye Lim Cho, Won Jun Kang

**Affiliations:** 1 Department of Nuclear Medicine, Chung-Ang University Hospital, Chung-Ang University College of Medicine, 102 Heukseok-ro, Dongjak-gu, Seoul 06973, Republic of Korea; soykim@cauhs.or.kr; 2 Department of Nuclear Medicine, Severance Hospital, Yonsei University College of Medicine, 50-1 Yonsei-ro, Seodaemun-gu, Seoul 03722, Republic of Korea; abies60@naver.com (J.Y.P.); etommi@yuhs.ac (Y.L.C.)

**Keywords:** fibroblast activation protein, [^68^Ga]Ga-FAPI-04, [^18^F]FDG, positron emission tomography, pulmonary fibrosis

## Abstract

**Background/Objectives:** Pulmonary fibrosis is a progressive and fatal lung disease with limited diagnostic and therapeutic options. Fibroblast activation protein (FAP) has emerged as a promising molecular imaging target for the non-invasive assessment of fibrotic activity. This study aimed to evaluate the diagnostic feasibility of [^68^Ga]Ga-FAP inhibitor (FAPI) and [^18^F]fluorodeoxyglucose ([^18^F]FDG) positron emission tomography (PET) for imaging pulmonary fibrosis in a mouse model. **Methods**: A pulmonary fibrosis model was established by intratracheal administration of polyhexamethylene guanidine-phosphate (PHMG-p) to C57BL/6 mice. Fibrosis severity was quantified by the Ashcroft scoring system using hematoxylin and eosin and Masson’s trichrome staining and evaluated by computed tomography (CT) imaging at 7, 14, and 21 days after PHMG-p exposure. PET imaging was performed, and ex vivo biodistribution was assessed after injection of [^68^Ga]Ga-FAPI-04 and [^18^F]FDG. **Results**: Histological analysis and Ashcroft scoring revealed greater fibrosis severity in the PHMG-p-treated group. Western blot analysis demonstrated upregulation of FAP expression after PHMG-p exposure. CT showed increased mean lung density, while [^68^Ga]Ga-FAPI-04 PET revealed significantly elevated pulmonary uptake of [^68^Ga]Ga-FAPI-04 in the PHMG-p-treated group compared with the controls. [^18^F]FDG PET imaging also showed higher uptake of [^18^F]FDG in the PHMG-p-treated group than in the controls. Ex vivo biodistribution confirmed greater [^68^Ga]Ga-FAPI-04 accumulation in the lungs of PHMG-p-treated mice. **Conclusions**: [^68^Ga]Ga-FAPI-04 PET serves as a sensitive imaging biomarker for evaluation of fibrotic activity in PHMG-p-induced pulmonary fibrosis and complements [^18^F]FDG PET for assessing disease progression and therapeutic response.

## 1. Introduction

Pulmonary fibrosis is a subset of interstitial lung diseases characterized by excessive deposition of extracellular matrix and scarring of the lung parenchyma, leading to progressive loss of respiratory function. Although the underlying etiologies vary, the most common form of pulmonary fibrosis is idiopathic pulmonary fibrosis (IPF), which has a poor prognosis, with five-year survival rates of 20–40% [1]. Treatment options for IPF are limited, and new therapeutic agents are currently under development [1,2,3]. Recently, new antifibrotic therapies such as nerandomilast have shown promising results in patients with IPF and progressive pulmonary fibrosis. These advances highlight the continued need for imaging biomarkers capable of assessing fibrotic activity and treatment response [4,5].

Computed tomography (CT) plays a crucial role in the diagnosis of pulmonary fibrosis, as it clearly depicts the structural patterns of fibrosis. However, conventional CT offers limited information about ongoing disease activity and metabolic or stromal remodeling [2,6]. Also, although histopathology provides details to make a definitive diagnosis, surgical lung biopsy involves considerable risk [7,8].

Therefore, several molecular imaging techniques have been developed to characterize fibrotic activity at different stages of the disease process. ^18^F-fluorodeoxyglucose ([^18^F]FDG) positron emission tomography (PET) has been explored as a marker of inflammatory activity, as it could correlate with disease severity and prognosis in IPF. However, [^18^F]FDG lacks specificity for fibrotic remodeling because the uptake reflects generalized glucose metabolism in inflammatory cells [9,10]. To overcome this limitation, fibrosis-specific molecular imaging probes have gained attention. For instance, integrin α_v_β_6_-targeted PET imaging visualizes early epithelial injury and transforming growth factor (TGF)-β activation [11], and collagen-binding radiopharmaceuticals such as [^68^Ga]CBP8 reflect extracellular matrix deposition which occurs later in the course of the disease [12]. Together, these approaches provide a complementary framework for assessing the dynamic nature of pulmonary fibrosis beyond structural CT findings.

Fibroblast activation protein (FAP) is selectively upregulated on activated stromal fibroblasts involved in tissue remodeling and scarring, making it an attractive imaging target [13,14]. In oncology, PET radiopharmaceuticals based on FAP inhibitors (FAPI) have shown high tumor-to-background contrast, consistent with the observation that FAP-positive cancer-associated fibroblasts are present in the stroma of a vast majority of epithelial tumors [15]. Early reports also suggest that FAPI PET may visualize fibro-inflammatory activity in non-oncologic diseases. However, systematic evaluation in pulmonary fibrosis remains relatively underexplored [16,17]. In a few studies, FAPI PET has demonstrated the ability to sensitively and noninvasively visualize fibroblast activation preceding morphologic fibrosis on CT. Uptake of FAPI-based radiopharmaceuticals correlated with histologic findings and treatment response, establishing FAPI PET as a promising biomarker for early detection and therapeutic monitoring in pulmonary fibrosis [16,17,18].

Given the heterogeneous causes of fibrosis, diverse animal models are required to reflect its varying pathogenic mechanisms. Bleomycin is the most widely used agent to induce pulmonary fibrosis in murine models [19]. However, it typically produces acute fibrosis and often resolves spontaneously over time, making it less suitable for studying chronic fibrotic disease. According to recent reports, lung injury and fibrosis can occur as a result of inhaling a humidifier disinfectant, polyhexamethylene guanidine phosphate (PHMG-p) [20,21,22]. In recent studies, PHMG-p has been widely used for studying progressive and irreversible pulmonary fibrosis [23,24,25].

In this study, we evaluated the diagnostic utility of [^68^Ga]Ga-FAPI-04 and [^18^F]FDG PET in a PHMG-p-induced pulmonary fibrosis mouse model. We hypothesized that FAPI PET, together with FDG PET, may provide a sensitive and fibrosis-specific readout of disease activity compared to the assessment of morphology alone.

## 2. Materials and Methods

### 2.1. Materials

All chemicals and solvents were purchased from Sigma-Aldrich (St. Louis, MI, USA) or Merck (Darmstadt, Germany) and were used without further purification. PHMG-p (25% solution, SKYBIO 1125, SK Chemicals) was provided by the Korea Institute of Toxicology (Jeongeup, Korea). The IGG-100 germanium-68/gallium-68 (^68^Ge/^68^Ga) generator was purchased from Eckert & Ziegler Radiopharma GmbH (1.85 GBq, Berlin, Germany). The DOTA-FAPI-04 was purchased from MedChemExpress LLC (Shanghai, China) with a purity of 98%. 4-(2-hydroxyethyl)piperazine-1-ethanesulfonic acid (HEPES, Cat# PHG0001) was purchased from GERBU Biotechnik GmbH (Heidelberg, Germany). The Chromafix^®^ 30-PS-HCO_3_ anion-exchange cartridge was purchased from Macherey-Nagel (Duren, Germany), and Sep-Pak C18 Plus Light cartridges were purchased from Waters (Milford, MA, USA). The 30-PS-HCO_3_ cartridge was pre-activated with 1 mL of 30% hydrochloric acid (HCl, Suprapur^®^ for trace analysis, Merck, Darmstadt, Germany), followed by washing with 10 mL deionized-distilled water (ddH_2_O) prior to use. Radio thin-layer chromatography (TLC) was performed using a glass microfiber chromatography paper impregnated with silica gel (iTLC-SG, Agilent Technologies, Santa Clara, CA, USA).

### 2.2. Animal Model of Pulmonary Fibrosis

Pulmonary fibrosis was induced in 6-week-old male C57BL/6 mice (Orient Bio Inc., Gyeonggido, Republic of Korea) by a single intratracheal instillation of PHMG-p (1.1 mg/kg) in 50 μL 0.9% NaCl solution. Mice were randomly assigned to either the control or PHMG-p-treated group to minimize selection bias. Randomization was performed using a computer-generated simple randomization list. Control mice received the same volume of 0.9% NaCl alone. The mice were housed in temperature (68–75 °F) and humidity (30–70%) controlled rooms under a 12 h light/dark cycle. All animal experimental procedures were reviewed and approved by the Animal Care Use Committee at Yonsei University (IACUC No. 2022-0091) and were performed according to the International Guide for the Care and Use of Laboratory Animals. This article is presented in accordance with the ARRIVE reporting checklist (Supplementary Materials).

### 2.3. Histology and Fibrosis Scoring

The left lungs of the mice were fixed in 4% paraformaldehyde, embedded in paraffin, and sectioned at 5 μm. After dewaxing and gradient ethanol hydration, the paraffin sections were stained with hematoxylin-eosin (H&E), and the histology was assessed by light microscopy (Olympus, Tokyo, Japan). The paraffin-embedded tissue sections were also stained using Masson’s trichrome kit according to the manufacturer’s instructions (Cat# ab150686; Abcam, Cambridge, MA, USA). The Ashcroft score was semi-quantitatively determined with Masson’s trichrome-stained sections under a microscope (Olympus) and analyzed at five points on each slide, as described previously [26]. The Ashcroft scoring was performed by an investigator blinded to group allocation.

### 2.4. Western Blot Analysis

The total proteins from the lung tissue specimens were extracted using a ProPrep Protein Extraction Solution (Cat# 17081; Intron Biotechnology, Inc., Seongnam, Republic of Korea) augmented with protease inhibitors, maintained on ice for 30 min. Cell lysates were subjected to clarification via centrifugation at 13,000× *g* for 20 min at 4 °C. Protein concentrations were quantified using the Pierce™ Bicinchoninic Acid (BCA) Protein Assay Kit (Cat# 23225; Thermo Fisher Scientific, Waltham, MA, USA). 40 µg of protein extract from each sample were separated using the Any kD™ Mini-PROTEAN^®^ TGX™ Precast Protein gel (Cat# 456-9033; Bio-rad Laboratories, Hercules, CA, USA) and transferred onto Immobilon polyvinylidene fluoride (PVDF) membranes (Cat# IPVH00010; MilliporeSigma, St. Louis, MO, USA). The membranes were blocked in Tris-Buffer Saline (TBS)-0.5% Tween 20 with 2% bovine serum albumin, and then incubated with the sheep polyclonal anti-human fibroblast activation protein alpha (FAP) antibody (Cat# AF3715; 1:400, R&D, Minneapolis, MN, USA) at 4 °C overnight and horseradish peroxidase (HRP)-conjugated secondary antibodies anti-sheep HAF016 (1:2000, R&D Systems, Minneapolis, MN, USA) at room temperature for 2 h. Signals were developed using Westar ETA C ULTRA2.0 (Cat# XLS075; Cyanagen, Bologna, Italy) and captured using the ImageQuant LAS 4000 mini system (GE Healthcare Bio-Sciences, Piscataway, NJ, USA).

### 2.5. Preparation of [^68^Ga]Ga-FAPI-04 and [^18^F]FDG

[^68^Ga]Ga-FAPI-04 was labeled with radioisotope Ga-68 according to the procedure described in a previous study [27,28] with slight modifications. Briefly, the [^68^Ga]GaCl_3_ was eluted from the ^68^Ge/^68^Ga generator with 5 mL of 0.1 M HCl and mixed with 4 mL of 30% HCl. The [^68^Ga]Ga^3+^ was trapped on the 30-PS-HCO_3_ cartridge and then eluted with ddH_2_O. The 25 μg of DOTA-FAPI-04 was diluted with 2 M HEPES solution, and the ^68^Ga-eluate was added (100 μL, 370–555 MBq). The final pH was about 3.85. The reaction mixture was incubated in an Eppendorf tube for 10 min at 95 °C. The product was isolated using a Sep-Pak C18 Plus Light cartridge and eluted with 0.9% NaCl/ethanol (1:1, *v*/*v*). The radiochemical purity of [^68^Ga]Ga-FAPI-04 was assessed by TLC on iTLC-SG strips as the stationary phase eluted with 1 M ammonium acetate in methanol (1:1) as the mobile phase. The iTLC-SG strips were scanned with a Bioscan AR-2000 radio-TLC scanner (Washington, DC) to determine the % area of radioactivity at the origin (representing free [^68^Ga]Ga) and solvent front (representing [^68^Ga]Ga-FAPI-04). The ^68^Ga-FAPI-04 was obtained at a radioactivity yield of 96.2 ± 1.1% with a total synthesis time of 20 min, and a radiochemical purity of >98%.

[^18^F]FDG was manufactured according to the standard method from mannose triflate with alkaline hydrolysis using the automated synthesis module (Neptis^®^ Perform, Philippeville, Belgium). The radiochemical purity of [^18^F]FDG was over 98%. The final product was sterile and pyrogen-free.

### 2.6. MicroCT and MicroPET Imaging

According to the results of the power analysis, three mice were included in each group for the imaging experiments. This sample size was considered sufficient to achieve a statistical power of 0.8. Mouse lung images were obtained using a Quantum GX2 micro-computed tomography (microCT) system (PerkinElmer, Waltham, MA, USA). Mice were intubated with a plastic tube and anesthetized by inhalation of 2% isoflurane. The microCT images were acquired using a respiratory-gated technique with the following parameters: X-ray tube voltage 90 kV, X-ray tube current 88 μA, a fixed filter of 0.5 mm aluminum, and 0.06 mm copper. The lungs were scanned throughout the 360° gantry rotation for 4 min, and a stack of 512 cross-sectional images with a voxel size of 50 µm was generated. Lung density was quantified in Hounsfield units (HU) by drawing regions of interest (ROI) in the lungs using the AW VolumeShare 7 software (GE Healthcare, version 4.7).

Small-animal PET images were obtained on an Inveon microPET scanner (Siemens, Knoxville, TN, USA). PHMG-p-treated mice were injected with [^68^Ga]Ga-FAPI-04 (7.4 MBq) or [^18^F]FDG (7.4 MBq) via the tail vein under 2% isoflurane anesthesia at 7, 14, and 21 days after PHMG-p exposure. The dynamic PET acquisitions were performed for 60 min immediately following a tracer injection. Data were acquired in the list mode format over 60 min (5 × 1 min frames, 11 × 5 min frames), and images were reconstructed using a three-dimensional ordered subsets expectation maximization (3D-OSEM) algorithm using the ASIPro VM™ Micro PET Analysis software (Siemens, version 6.2.5.0). Static PET images were reconstructed by averaging frames acquired 30–40 min after FAPI injection and 50–60 min after FDG injection. An elliptical ROI was manually drawn on the right lung parenchyma while carefully avoiding the cardiac area. Quantification was restricted to the right lung because high myocardial uptake leads to pronounced spillover into the adjacent left lung in microPET, resulting in inconsistent and unreliable measurements. Mean standardized uptake values (SUVmean) were calculated from the ROIs using the AMIDE software (version 1.0.6).

### 2.7. Assessment of Ex Vivo Biodistribution

PHMG-p-treated mice were injected with [^68^Ga]Ga-FAPI-04 (7.4 MBq) or [^18^F]FDG (7.4 MBq) via the tail vein under 2% isoflurane anesthesia. PHMG-p-treated mice were sacrificed at 30 min after injection of [^68^Ga]Ga-FAPI-04 and 1 h after injection of [^18^F]FDG (*n* = 4 for each group). Major organs (lung, heart, liver, spleen, kidney, stomach, small intestine, large intestine, muscle, femur) were harvested and weighed. Blood samples were collected by cardiac puncture under 5% isoflurane anesthesia. The radioactivity of each sample was measured using a 2470 Wizard^2^ automatic gamma counter (PerkinElmer, Waltham, MA, USA). For comparison, 1% injected activity standards were prepared and counted along with the samples. Radioactivity concentration was expressed as a percentage of the injected dose per gram of tissue (%ID/g).

### 2.8. Statistical Analysis

The data and statistical evaluations were conducted using GraphPad version 7.0 (Prism). Quantitative data are presented as mean ± standard deviation (SD). *p* values were computed using ANOVA analysis, with a *p* < 0.05 threshold deemed statistically significant.

## 3. Results

### 3.1. FAP Expression in the PHMG-p-Induced Pulmonary Fibrosis Model

**Figure 1:**
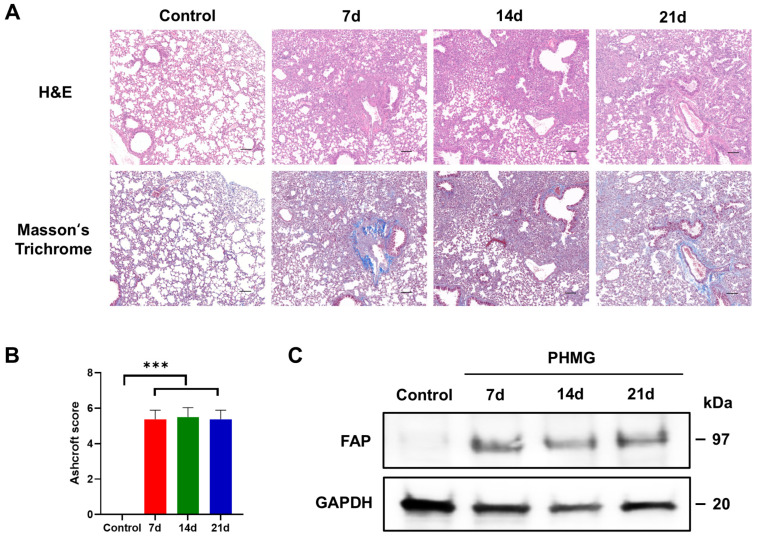
Polyhexamethylene guanidine-phosphate (PHMG-p) induced progression of lung fibrosis and expression of fibroblast activation protein (FAP) in vivo. (**A**) Histological analysis to evaluate the severity of pulmonary fibrosis after PHMG-p administration. Representative images after hematoxylin and eosin (H&E) and Masson’s trichrome staining of the lung tissues of the control and PHMG-p-treated group on days 7, 14, and 21. Scale bar = 100 μm (**B**) Quantitation of lung fibrosis using the Ashcroft score following PHMG-p exposure. Data are expressed as mean ± SD. Statistical significance was confirmed by one-way ANOVA with Tukey post hoc analysis. *** *p* < 0.001 versus the untreated controls. (**C**) Representative Western blot images of FAP levels in lungs of PHMG-p-treated mice.

The development of pulmonary fibrosis was confirmed by H&E and Masson’s trichrome staining of the left lung tissue of the mice. Histological analysis of the lung tissues revealed fibrotic changes after exposure to PHMG-p. H&E staining showed alveolar wall thickening, architectural distortion, and extensive infiltration of inflammatory cells in the PHMG-p-treated group compared to the control group (Figure 1A). Masson’s trichrome staining confirmed increased collagen deposition in the interstitial and peribronchial regions compared to the control group (Figure 1A). Semi-quantitative analysis using the modified Ashcroft fibrosis scoring revealed that the degree of pulmonary fibrosis in the PHMG-p-treated group was significantly higher than that in the control group (Figure 1B). Mean Ashcroft scores were 0.00 ± 0.00 in controls, 5.38 ± 0.18 at 7 days, 5.50 ± 0.19 at 14 days, and 5.38 ± 0.18 at 21 days. One-way ANOVA confirmed a highly significant group effect (*p* < 0.01). Post hoc Tukey testing revealed that all the mice in the PHMG-p-treated group had significantly higher scores than the control group (*p* < 0.001), while no significant differences were observed within the PHMG-p-treated group. These results showed that pulmonary fibrosis was successfully induced within 7 days after intratracheal administration of PHMG-p.

To determine whether PHMG-p induces abnormal activation of FAP, we measured the expression levels of the FAP using Western blot analysis. PHMG-p elevated FAP expression in the lung homogenate of PHMG-p-treated mice compared to the control group, with sustained expression observed across 7, 14, and 21 days (Figure 1C).

### 3.2. CT Imaging of Lung Injury in the PHMG-p-Induced Pulmonary Fibrosis Model

**Figure 2:**
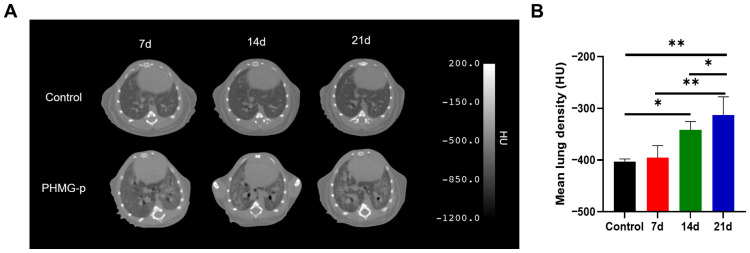
CT imaging of the lungs of mice in PHMG-p-induced pulmonary fibrosis. (**A**) Representative serial CT images of the control and PHMG-p-treated groups on days 7, 14, and 21. (**B**) Quantitative analysis of lung density by microCT. Lung density was quantified in Hounsfield units (HU). Data are expressed as mean ± SD. Statistical significance was confirmed by one-way ANOVA with Tukey post hoc analysis. * *p* < 0.05, ** *p* < 0.01.

The microCT images showed enhanced ground-glass opacity, consolidation, and bronchiectasis in the lungs of mice after intratracheal instillation of PHMG-p (Figure 2A). The mean lung density of the PHMG-p-treated group (−395.50 ± 21.00 HU) was slightly higher than the control group (−403.30 ± 5.00 HU) on day 7. However, after 14 days of PHMG-p treatment, the PHMG-p-exposed group showed significantly higher Hounsfield unit values in the lung tissue than the control group (*p* < 0.05) (Figure 2B).

### 3.3. In Vivo PET Imaging of [^68^Ga]Ga-FAPI-04 in the PHMG-p-Induced Pulmonary Fibrosis Model

**Figure 3:**
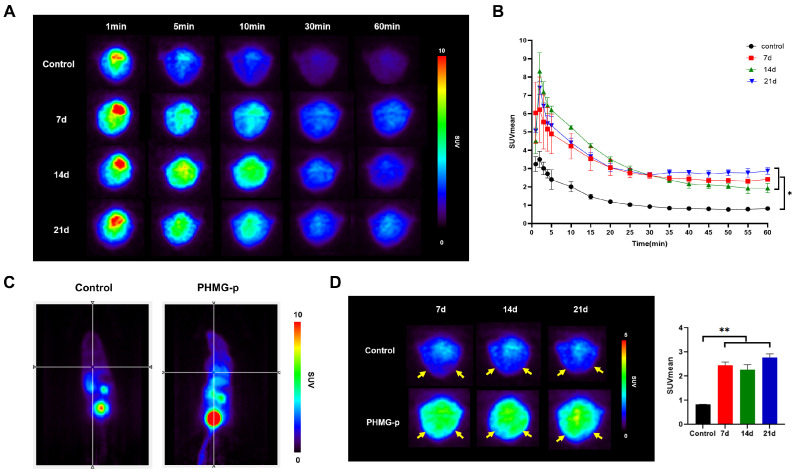
[^68^Ga]Ga-FAPI-04 PET imaging of PHMG-p-induced pulmonary fibrosis model (**A**) Representative axial PET images of lung at 1, 5, 10, 30, and 60 min post-injection of [^68^Ga]Ga-FAPI-04. (**B**) Time–activity curves of [^68^Ga]Ga-FAPI-04 uptake in the lung. * *p* < 0.05 (**C**) Representative coronal PET images of the control and PHMG-p-treated mice at 30 min post-injection of [^68^Ga]Ga-FAPI-04 on day 14. (**D**) Static PET images of the lung (yellow arrow) at 30 min post-injection of [^68^Ga]Ga-FAPI-04 on days 7, 14, and 21 after instillation of PHMG-p. Comparative quantitative analysis of [^68^Ga]Ga-FAPI-04 uptake in the lungs of the control and PHMG-p-treated mice. Data are expressed as mean ± SD. Statistical significance was confirmed by one-way ANOVA with Tukey post hoc analysis. ** *p* < 0.01.

Dynamic [^68^Ga]Ga-FAPI-04 PET imaging demonstrated different pulmonary uptake patterns between the control and PHMG-p-treated groups (Figure 3A). In the control group, [^68^Ga]Ga-FAPI-04 uptake declined rapidly within the first 10 min and remained low thereafter. In the PHMG-p-treated group, uptake of [^68^Ga]Ga-FAPI-04 was initially elevated compared to the controls and declined rapidly but remained persistently higher than the control group throughout the 60 min dynamic acquisition (Figure 3B). Since the pulmonary uptake of [^68^Ga]Ga-FAPI-04 approaches a kinetic plateau approximately 30 min post-injection, a static PET image was reconstructed by averaging the dynamic frames acquired between 30 and 40 min. The control mice showed minimal retention of pulmonary tracer with low background activity. In contrast, the PHMG-treated mice exhibited markedly increased uptake in the lung fields (Figure 3C). On days 7, 14, and 21 after treatment with PHMG-p, the PHMG-p-treated group showed significantly higher [^68^Ga]Ga-FAPI-04 uptake in the lungs compared to the control group (*p* < 0.05) (Figure 3D). No significant differences were found within the PHMG-p-treated group.

### 3.4. In Vivo PET Imaging of [^18^F]FDG in PHMG-p-Induced Pulmonary Fibrosis Model

**Figure 4:**
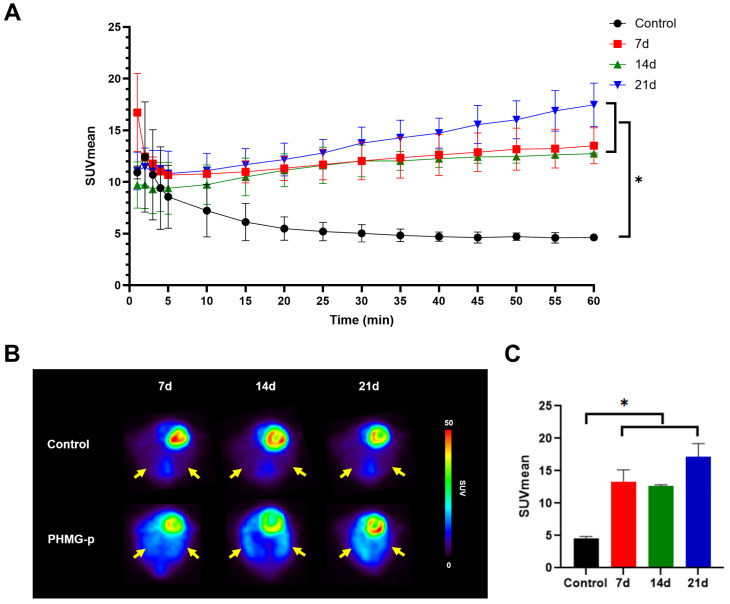
[^18^F]FDG PET imaging of PHMG-p-induced pulmonary fibrosis model. (**A**) Time–activity curves of [^18^F]FDG uptake in the lung. * *p* < 0.05 (**B**) Representative transverse PET images of lung (yellow arrow) obtained at days 7, 14, and 21 after instillation of PHMG-p. In vivo [^18^F]FDG PET images were acquired at 50–60 min post-injection of [^18^F]FDG (**C**). Comparison of pulmonary [^18^F]FDG uptake (SUV_mean_) of the control and PHMG-p-treated mice. Data are expressed as mean ± SD. Statistical significance was confirmed by one-way ANOVA with Tukey post hoc analysis. * *p* < 0.05.

[^18^F]FDG PET imaging also showed distinct differences in uptake patterns between the control and the PHMG-p-treated group (Figure 4A). In the control mice, the pulmonary uptake of [^18^F]FDG rapidly decreased within the first 10 min and reached a stable plateau thereafter. In contrast, the PHMG-p-treated group (7 d, 14 d, and 21 d) exhibited persistently higher uptake throughout the dynamic acquisition. After 20 min, all PHMG-p-treated groups displayed a significantly higher uptake than the controls (*p* < 0.05), and this difference persisted until 60 min. However, no significant differences were observed within the PHMG-p-treated group. Static PET images were reconstructed by averaging dynamic frames acquired between 50 and 60 min (Figure 4B). PHMG-p-treated groups showed significantly higher [^18^F]FDG uptake compared with the control group (*p* < 0.05) (Figure 4C).

### 3.5. Ex Vivo Biodistribution in PHMG-p-Induced Pulmonary Fibrosis Model

**Figure 5:**
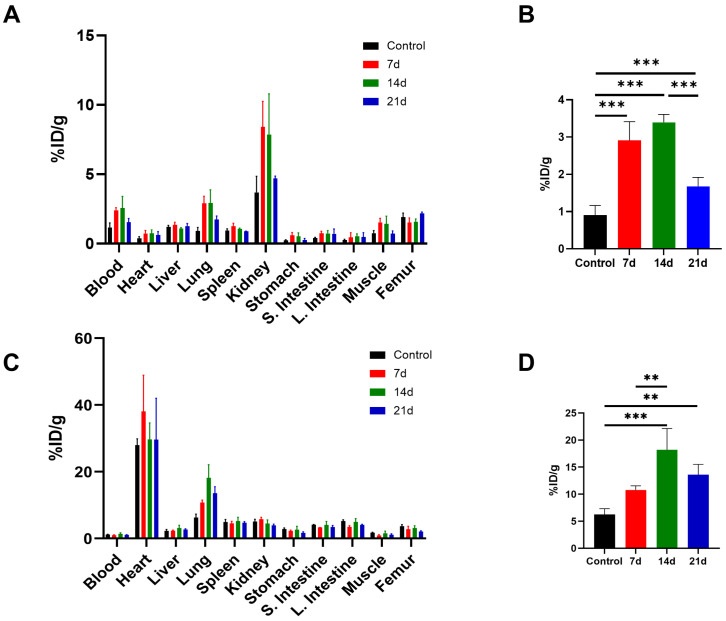
Comparison of the ex vivo biodistribution. (**A**) Biodistribution of [^68^Ga]Ga-FAPI-04 was assessed in PHMG-p-treated mice. (**B**) Comparison of the pulmonary uptake of [^68^Ga]Ga-FAPI-04 in the control and PHMG-p-treated mice. (**C**) Biodistribution data for [^18^F]FDG. (**D**) Comparison of the lung [^18^F]FDG uptake of control and PHMG-p-treated mice. The organ uptake values were expressed as the percentage of the injected dose per gram (%ID/g). Data are expressed as mean ± SD. Statistical significance was confirmed by one-way ANOVA with Tukey post hoc analysis. ** *p* < 0.01, *** *p* < 0.001.

To quantitatively analyze the uptake of [^68^Ga]Ga-FAPI-04 within the lung, the ex vivo distribution was assessed on days 7, 14, and 21 after instillation of PHMG-p. The biodistribution of [^68^Ga]Ga-FAPI-04 at 30 min after injection is shown in Figure 5A. Consistent with the PET findings, [^68^Ga]Ga-FAPI-04 rapidly washed out and was primarily excreted through the renal system in vivo. Biodistribution analysis revealed a significant increase in the pulmonary uptake of [^68^Ga]Ga-FAPI-04 after the PHMG-p exposure compared with the controls (*p* < 0.001). Mean lung uptake was 0.91 ± 0.26 %ID/g in the controls, and 2.91 ± 0.51 %ID/g, 3.40 ± 0.21 %ID/g, and 1.68 ± 0.24 % ID/g at 7, 14, and 21 days after PHMG-p treatment, respectively (Figure 5B). [^68^Ga]Ga-FAPI-04 uptake peaked at 14 days, which was significantly greater than that at 21 days (*p* < 0.001). These results suggest that pulmonary retention of [^68^Ga]Ga-FAPI-04 increases after PHMG-p exposure, reaches a maximum at 14 days, and partially decreases by 21 days but remains higher than in the control group.

Ex vivo biodistribution of [^18^F]FDG was assessed in the PHMG-p-treated mice at 60 min after injection (Figure 5C). The mean lung uptake of [^18^F]FDG was 6.28 ± 1.05 %ID/g in controls, 10.78 ± 0.77 %ID/g, 18.19 ± 3.95 %ID/g, and 13.60 ± 1.92 %ID/g at 7, 14, and 21 days after PHMG-p treatment, respectively (Figure 5D). [^18^F]FDG uptake was higher at 14 days than in the control (*p* < 0.001) and 7-day groups (*p* = 0.008), and higher at 21 days than in controls (*p* = 0.005), while the 7-day group did not differ from controls. Similarly to the biodistribution results of [^68^Ga]Ga-FAPI-04, [^18^F]FDG uptake in the lungs peaked at 14 days after the PHMG-p administration. These findings indicate that pulmonary [^18^F]FDG retention is also increased following PHMG-p exposure.

## 4. Discussion

In this study, we demonstrated that [^68^Ga]Ga-FAPI-04 and [^18^F]FDG sensitively capture fibroblast activation in a PHMG-p-induced pulmonary fibrosis murine model, thereby complementing CT, which provides anatomic but not functional information. IPF remains a chronic and devastating lung disease, characterized by progressive fibrotic remodeling with limited treatment options and poor prognosis [2]. Accurate and noninvasive assessment of disease activity is critical for improving clinical outcomes. Therefore, molecular imaging represents a promising strategy to address this need [29]. Several molecular imaging strategies have been developed to characterize fibrotic activity, each reflecting distinct stages of the fibrogenic cascade. FAP is a type II transmembrane serine protease minimally expressed in normal adult tissues but strongly upregulated in fibrotic lesions, chronic inflammation, and cancer-associated fibroblasts. Targeting FAP with FAPI-based radiopharmaceuticals thus enables direct imaging of fibroblast activation, the core driver of fibrogenesis [30].

Among the various FAPI-based radiopharmaceuticals, [^68^Ga]Ga-FAPI-04 is widely used due to its ease of synthesis, favorable biodistribution, and versatility for theranostic applications. [^68^Ga]Ga-FAPI-04 was chosen for this study because it is the most validated compound, with well-established radiolabeling procedures and reproducible imaging characteristics [31,32].

In our study, FAP expression was increased in the PHMG-p-treated group as observed on the Western blot, consistent with previous reports demonstrating increased FAP expression in pulmonary fibrosis [33]. H&E staining demonstrated fibrotic changes, and Masson’s trichrome staining confirmed collagen deposition in the PHMG-p-treated groups, suggesting that [^68^Ga]Ga-FAPI-04 uptake is closely associated with histological fibrosis. The Ashcroft score was also significantly higher in the PHMG-p-treated group when compared with the control group. However, the Ashcroft score was similar among the PHMG-p-treated groups. The inherent limitations of the Ashcroft scoring system can explain this. As a semi-quantitative method, it relies on categorical grading (0–8) rather than continuous measurement, which reduces its sensitivity to subtle changes in fibrosis. In addition, the scoring is observer-dependent, leading to potential inter- and intra-observer variability [34]. These limitations emphasize the necessity of quantitative imaging modalities for more sensitive assessment of pulmonary fibrosis. Ex vivo biodistribution confirmed the imaging results, with increased pulmonary [^68^Ga]Ga-FAPI-04 and [^18^F]FDG uptake in the PHMG-p-treated groups. This supports the value of [^68^Ga]Ga-FAPI-04 and [^18^F]FDG as promising biomarkers sensitive to fibrotic activity.

Preclinical studies in mouse models of pulmonary fibrosis have demonstrated that FAPI PET enables sensitive detection of early fibrotic changes [16,17,18]. Rosenkrans et al. showed that [^18^F]FDG and [^68^Ga]Ga-FAPI-46 successfully detected fibrotic activity as early as 6 and 7 days, respectively, following bleomycin instillation in a murine model, whereas CT revealed significant changes only at day 14 [17]. Consistent with these findings, our study also showed that CT detected significant differences compared to the controls from day 14 onward, while both [^18^F]FDG and [^68^Ga]Ga-FAPI-04 PET showed elevated uptake as early as day 7.

Several studies have demonstrated that [^18^F]FDG activity correlates with the severity and prognosis of IPF. While the exact cellular mechanisms underlying [^18^F]FDG uptake in IPF remain unclear, upregulation of glucose transporter 1 has been observed in fibroblasts and bronchial epithelial cells during the fibrotic process. However, [^18^F]FDG reflects glucose metabolism in inflammatory and immune cells as well as fibrosis [9,35,36,37]. Previous studies have consistently shown that [^18^F]FDG PET predominantly reflects inflammatory processes [36,38,39]. Therefore, compared with FAPI PET, which more specifically represents fibrotic activity, [^18^F]FDG PET demonstrates lower specificity [40].

In this study, the partial washout observed in the dynamic [^68^Ga]Ga-FAPI-04 PET curve in the PHMG-p-treated groups supports the notion that [^68^Ga]Ga-FAPI-04 uptake reflects reversible binding to FAP expressed on activated fibroblasts, not to nonspecific lesions, consistent with fibroblast-specific targeting. In contrast, the uptake of [^18^F]FDG remains stable over time without washout in the [^18^F]FDG PET curve. This indicates that [^18^F]FDG uptake includes nonspecific glucose metabolism in metabolically active inflammatory and epithelial cells, as well as fibroblast cells, because [^18^F]FDG is irreversibly trapped after phosphorylation [41,42]. To the best of our knowledge, no previous studies have specifically reported time–activity curves from dynamic [^68^Ga]Ga-FAPI-04 PET in pulmonary fibrosis.

Recent studies highlight that [^18^F]FDG PET is most effective in the inflammatory phase, while FAPI PET outperforms it in detecting ongoing fibrosis and monitoring disease progression and therapeutic response to antifibrotic drugs such as pirfenidone and nintedanib [16,17]. Uptake of FAPI-based radiopharmaceuticals correlates closely with fibrotic burden and response to antifibrotic treatments [16,18]. Also, early human studies have reported high FAPI-based radiotracer uptake in fibrotic interstitial lung disease, suggesting the feasibility of clinical translation [43,44]. Given the poor prognosis and lack of reliable non-invasive biomarkers in IPF, FAPI PET holds promise for assessing disease activity and monitoring the therapeutic response in patients.

Pulmonary fibrosis encompasses a heterogeneous group of interstitial lung diseases characterized by excessive extracellular matrix deposition and the architectural distortion of lung parenchyma. IPF is the most common form of pulmonary fibrosis. However, many other forms also exist, including environmental and occupational forms caused by silica or asbestos exposure, drug- or toxin-induced fibrosis, connective tissue disease–associated fibrosis, and radiation-induced fibrosis [45]. Because each subtype exhibits distinct etiologies, inflammatory responses, cellular activation pathways, and progression dynamics, no single experimental model can fully recapitulate the complexity of human pulmonary fibrosis. For this reason, various murine models have been established to reflect the diverse pathogenic mechanisms of this disease [19,46]. Although the bleomycin-induced pulmonary fibrosis model is the most widely used, it predominantly induces acute lung injury followed by partially reversible fibrosis, which may limit its ability to fully reproduce the chronic and progressive nature of human IPF [47,48]. In contrast, PHMG-p exposure results in persistent inflammatory responses and sustained fibrotic remodeling, as demonstrated by histopathological studies, biomarker analyses, and longitudinal imaging correlations [49,50]. To our knowledge, this is the first study to evaluate PET/CT in PHMG-p-induced pulmonary fibrosis.

This study has certain limitations. The modest sample size limits its power for detecting subtle differences between the PHMG-p-treated groups. PHMG-p-induced fibrosis in mice may not fully replicate the complex heterogeneity of human IPF [25]. PHMG-p–induced fibrosis represents a preclinical model that recapitulates certain pathological features of fibrotic lung injury; however, it does not fully mirror the nature of human IPF. Therefore, our findings should be interpreted as demonstrating the potential feasibility of FAPI and FDG PET for assessing fibroinflammatory activity in an experimental setting, rather than implying direct clinical equivalence. Extrapolation to human IPF is inherently limited, and further validation in patients with IPF is required to establish clinical relevance. Notably, extra-pulmonary [^68^Ga]Ga-FAPI-04 uptake is observed in the heart. Further evaluation is needed to evaluate systemic PHMG-p toxicity or fibro-inflammatory remodeling beyond the lungs [51,52,53].

## 5. Conclusions

In summary, [^68^Ga]Ga-FAPI-04 provided sensitive and fibrosis-specific imaging of pulmonary fibrosis, while [^18^F]FDG offered complementary information on inflammatory metabolism. [^68^Ga]Ga-FAPI-04 could be a promising molecular imaging biomarker for evaluating disease progression and treatment response in pulmonary fibrosis.

## Data Availability

The raw data supporting the conclusions of this article will be made available by the authors on request.

## Supplementary Materials

The following supporting information can be downloaded at: https://www.mdpi.com/article/10.3390/diagnostics16010010/s1, Supplementary File S1: ARRIVE Checklist.
